# Maxwell A. Pollack Award Lecture

**DOI:** 10.1093/geroni/igab046.1291

**Published:** 2021-12-17

**Authors:** Karl Pillemer

**Affiliations:** Cornell University, Ithaca, New York, United States

## Abstract

The lecture will feature an address by the 2020 Pollack Award recipient, Karl Pillmer, PhD, FGSA of Cornell University. The 2021 Pollack Award recipient is Namkee G. Choi, PhD, FGSA, of the University of Texas at Austin. The Maxwell A. Pollack Award for Contributions to Healthy Aging Award recognizes instances of practice informed by research and analysis, research that has directly improved policy or practice, and distinction in bridging the worlds of research and practice.

